# Molecular Targets of β-Lactam-Based Antimicrobials: Beyond the Usual Suspects

**DOI:** 10.3390/antibiotics3020128

**Published:** 2014-04-03

**Authors:** Monika I. Konaklieva

**Affiliations:** Department of Chemistry, American University, 4400 Massachusetts Ave. NW, Washington, DC, 20016-8014, USA; E-Mail: mkonak@american.edu; Tel.: +1-(202)-885-1777; Fax: +1-(202)-885-1752

**Keywords:** antibiotic resistance, directed evolution, β-lactams as chemical probes

## Abstract

The common practice in antibacterial drug development has been to rapidly make an attempt to find ever-more stable and broad-spectrum variants for a particular antibiotic, once a drug resistance for that antibiotic is detected. We are now facing bacterial resistance toward our clinically relevant antibiotics of such a magnitude that the conversation for antimicrobial drug development ought to include effective new antibiotics with alternative mechanisms of action. The electrophilic β-lactam ring is amenable for the inhibition of different enzyme classes by a suitable decoration of the core scaffold. Monocyclic β-lactams lacking an ionizable group at the lactam nitrogen exhibit target preferences toward bacterial enzymes important for resistance and virulence. The present review intends to draw attention to the versatility of the β-lactams as antimicrobials with “unusual” molecular targets.

## 1. Introduction

### Ingenuity of the β-Lactam as the Acylating Agent

**Figure 1:**
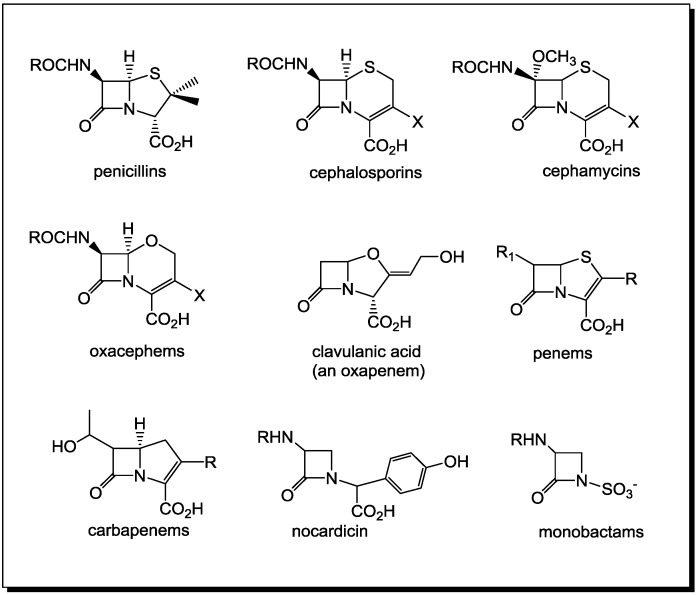
Clinically relevant β-lactam antibiotics.

β-Lactams occur relatively rarely in nature. Initial work by Strominger indicated that the activity of penicillin was due to the inherent strain of the four-membered ring or to the reduced amide resonance [1,2]. However, many amide and lactam derivatives are as chemically reactive as the penicillins and cephalosporins [3,4]. This raises the question of what factors account for the seemingly special nature of β-lactam antibiotics as compared to other acylating agents [5,6,7,8]. A shift in the understanding of the biological activity of β-lactams from the traditional view of penicillins as effective acylating agents, to the necessity for a proper molecular recognition between the lactam or amide and its host protein has occurred [3,9]. Apparently, all β-lactams with a current therapeutic application operate by means of mechanisms resulting in the formation of hydrolytically stable enzyme complexes. Derived biochemically from two molecules of l-cysteine, penicillin has been designed by fungi to mimic the d-alanine-d-alanine termini of bacterial peptidoglycans, which allows it to be recognized by transpeptidases. The latter interlinks peptidic residues between peptidoglycan strands in the bacterial cell wall [10]. This crucial step for securing the integrity of the bacterial cell wall is interrupted by penicillin, which irreversibly acylates the active site serine of the transpeptidases. For “classical” bicyclic β-lactam antibiotics, (Figure 1) the antibacterial activity is due to the position of the lactam nitrogen in the ring fusion, which allows for sufficient pyramidalization of the nitrogen center, which, in turn, perturbs the resonance stabilization of the lactam amide. Therefore, the *N*-fused bicyclic β-lactams have enhanced lactam electrophilicity toward nucleophilic ring opening, easily reacting with nucleophilic amino acid side chain functionalities, including those essential to proper enzyme function. Recently, it has been hypothesized that the acylating ability of the penicillin-like β-lactam antibiotics, approximated to that of acid chlorides [2,11], is aided by an intramolecular protonation of the lactam nitrogen by the neighboring carboxylic acid residue within the active site of the transpeptidases [12].

Based on experimental data obtained from inelastic neutrons and quantum chemical theory, it has been suggested that penicillin changes from relatively inactive at near neutral physiological pH, to a highly electrophilic amide in the acidic active site environment [12]. This shift in reactivity could explain the ability of penicillin to travel to its molecular target, the bacterial cell wall transpeptidases, in the mammalian organism unaltered and demonstrate yet again the cleverly designed weapon at the molecular level—the appropriately substituted β-lactam as the acylating reagent. The hydrolysis rate of unsubstituted 2-azetidinone is considerably slower in acidic media and is virtually unchanged under basic conditions compared to that of the penicillin [12].

In 1981, two independent groups from the Squibbs and Takeda laboratories reported the isolation of the first *N*-thiolated β-lactams derived from natural sources. These β-lactams differed from all previously reported antibiotics by having a monocyclic ring with an *N*-sulfonic acid group attached directly to the nitrogen (Figure 1). They named these “monobactams” to highlight, for the first time, that monocyclic β-lactams are present in the environment and have potent antimicrobial activities. Aztreonam, the first marketed monobactam, has activity against most aerobic Gram-negative bacilli, including *Pseudomonas aeruginosa*. These reports confirmed that β-lactams do not strictly require a conformationally constrained bicyclic ring structure to possess antibacterial properties. These monobactams display a broad spectrum of activity against aerobic Gram-negative bacteria, but little or no activity against Gram-positive bacteria, such as *Staphylococcus aureus*.

Recently, Csizmadia and co-workers defined a new “amidicity” index, which is used to quantify the relative amide character for a wide range of amides [13,14,15]. Their method utilizes computed enthalpies of the hydrogenation of the amide carbonyls, which should reflect the degree of amide character. In addition, two independent computational methods have been used by Glover and co-workers for the determination of the amidicity of a range of amides, including β-lactams [16]. In the latter work and elsewhere, monocyclic β-lactams have been computed to be planar at the nitrogen, but bicyclic systems in the penam/em and cepham/em scaffolds have varying degrees of twists about the lactam C−N bond and pyramidalization at the nitrogen, which, with the exception of the cepham system, result in reduced amidicities relative to *N,N*-dimethylacetamide (the archetypical amide). It is interesting to note that these computational results indicate that the loss of amidicity, even in the highly pyramidal penam and penem scaffolds, is not excessive, which accounts for the stability of β-lactam antibiotics to side reactions in the transport of β-lactam antibiotics to their target enzymes. Moreover, since correlations of reactivity with factors, such as pyramidality and, therefore, amidicity alone, have been determined to be poor, this supports the view that the transport, metabolism and target binding characteristics of β-lactam antibiotics must play the dominant role in their biological activity [3,9].

## 2. PBPs and β-Lactamases: the Two Main Molecular Targets for Drug Development, So Far

For more than seven decades, penicillins and related antibiotics have been used widely for the control and treatment of bacterial infections [17,18]. As many as 40 structurally different β-lactam compounds in 73 formulations are currently available for medical use. Improving upon the effectiveness of this class of antimicrobial agents has been an ongoing challenge, one which has continued to attract increasing attention, because of the emergence of multidrug-resistant strains of bacteria [19,20,21,22,23,24]. Over the years, countless penicillin derivatives [25,26,27] have been prepared and tested, and a variety of new β-lactam ring systems have been introduced (Figure 1). These systems include the penems, cephalosporins, carbapenems, oxapenams, oxacephams, as well as monocyclic, spirocyclic and multicyclic ring systems.

At least three families of bacterial enzymes specifically recognize β-lactam antibiotics. These include transpeptidase enzymes, or penicillin binding proteins (PBPs), which are the inhibitory targets for antibiotics, β-lactam synthases, the enzymes that biosynthesize penicillin, cephalosporins and monobactams, and β-lactamases, the defense enzymes of many drug-resistant bacteria. β-Lactamases are generally divided into serine- and zinc-dependent enzymes [28]. PBPs and Class A, C and D β-lactamases have an active-site serine, a property they share with a large class of enzymes known as serine proteases. Class B represents the bacterial metallo-β-lactamases.

In 1976, Jean-Marie Frère and coworkers [29] suggested that an “active site” model of the PBPs can be represented kinetically:

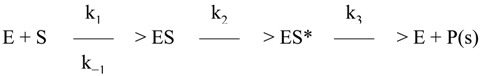

where E is the enzyme, S the antibiotic, ES a non-covalent complex, ES* a covalent acyl-enzyme and P(s) the inactivated product(s) of degradation of the antibiotic. Efficient inactivation of the enzyme depends on the rapid and nearly quantitative accumulation of the ES* complex, which is the result both of its stability (a low k_3_ value) and its rapid formation (generally due to high k_2_ values).

In subsequent years, it became clear that the above model depicts how β-lactams interact not only with PBPs, but also with a large number of β-lactamases, involving in all cases the initial acylation of the active site serine [30]. Plasmid-mediated production of β-lactamases is largely responsible for the resistance of many bacteria to the normally lethal action of β-lactam antibiotics. There has been much discussion on the evolutionary relationship between β-lactamases and transpeptidases [31,32,33,34,35,36]. Several reviews on the kinship between PBPs and serine β-lactamases [36,37,38], which belong to the superfamily of β-lactam recognizing enzymes [34,39,40,41,42], have appeared in the literature. It is the more rapid deacylation rate of the β-lactamases that separates them from the PBPs. Thus, nature appears to have taken a basic conserved protein template and, through mutation and selection, to have produced two types of bacterial enzymes. These enzymes differ from one another, but both have vital functions for the survival of the bacteria. There is little similarity between the PBPs, the serine β-lactamases and the rest of the serine enzymes, either structurally or with regard to amino acid (AA) sequence [43,44,45,46].

**Figure 2:**
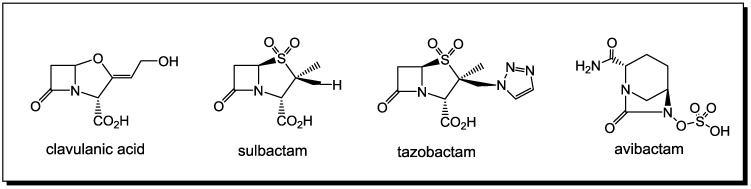
Clinically relevant β-lactamase inhibitors.

Although reports emerged as early as 1940 that some strains of bacteria can exhibit resistance to penicillin, this had no clinical relevance until the 1970s [47,48]. Today, resistance to antibiotics is a global crisis [49,50,51,52] with multiple drug resistance (MDR) and extreme drug resistance (XDR) reported in both community and healthcare settings [53]. Bacterial resistance to β-lactams generally operates by three different mechanisms: decreased access of antimicrobials to the target PBPs (efflux pumps), altered PBPs (affinity of binding decreased) and β-lactamase production. The latter is by far the most efficient of the resistance mechanisms [54]. Two strategies have been used against the action of the β-lactamases. One is the design of β-lactams that are poorer substrates for many common β-lactamases, including the so-called extended spectrum β-lactamases, whose distribution in nature is expanding. These agents include the third-generation cephalosporins, the carbapenem, imipenem, and the monobactam, aztreonam. The other is the design of β-lactamase inhibitors. The search for β-lactamase inhibitors has led to the identification of clavulanic acid, tazobactam and sulbactam [55] (Figure 2), which have been introduced into the market in formulations [56] combining them with a variety of β-lactam antibiotics (inhibitors of PBPs) [57].

Currently, the majority of clinically relevant β-lactamase inhibitors have activity against the Class A β-lactamases, the most common class at this time [37,38,58]. Recently, inhibitors such as avibactam, currently in Phase III clinical trials with activity against Class C and D β-lactamases, have been reported [59]. The structures of numerous potential β-lactamase inhibitors have been synthesized and tested in the last few decades [58,59,60,61,62]. The history, microbiology, synthesis and mechanism of the inhibition of the β-lactamases have all been reviewed [5,6,7,8,58,63,64,65,66].

## 3. β-Lactams Lacking the Ionizable Residue at the Lactam Nitrogen: A New Direction of Antimicrobial Compounds against Bacteria

All of the clinically relevant β-lactam antibiotics and those in clinical trials [67,68] contain an ionizable group either in the proximity (carboxylic acid, bicyclic, penicillin-like structures) or on (sulfonic acid, monobactams) the lactam nitrogen of the β-lactam ring. Until recently, it was generally accepted that for β-lactams to exert bactericidal activity, they must contain a scaffold, which specifically has an ionizable group at the lactam nitrogen within 3.6 Å of the β-lactam carbonyl carbon. However, there now appear to be exceptions to this scaffold requirement, since *N*-alkylthiolated β-lactams possess inhibitory, although not cidal, antimicrobial activity [69]. Subsequent reports have confirmed that once that ionizable group is “removed” from the lactam nitrogen, a variety of novel molecular targets begin to emerge [70,71,72].

**Figure 3:**
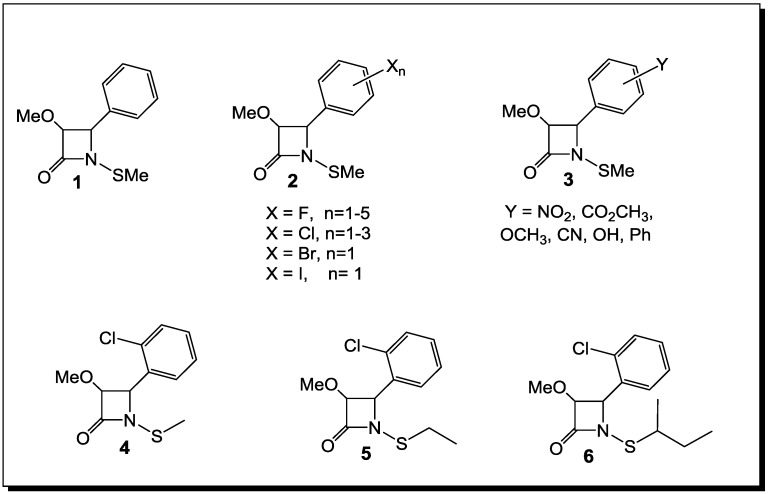
*N*-alkylthio β-lactams active against *Staphylococcus* spp*.* (including methicillin-resistant *S. aureus* (MRSA)) and *Bacillus* spp*.*

The synthesis and the biological evaluation as antibacterial agents of monocyclic β-lactams with an alkylthio group on the lactam nitrogen [69,73,74,75,76], stable to the hydrolytic activity of β-lactamases (Figure 3), have been reported in the literature in the last decade. The initial studies of *N*-thiolated β-lactams have focused on the determination of their structure-activity toward *Staphylococcus*, especially methicillin-resistant *S. aureus* strains (MRSA). There are two important observations that have come from this work, so far: (1) the ring functionality does have a role, albeit still a rather undefined one, on the *in vitro* anti-MRSA properties of the β-lactams; and (2) most significantly, the *N*-organothio moiety is an absolute requirement for bioactivity. To extend these investigations further, the *in vitro* activity of the more promising compounds against *S. aureus* have been evaluated for a much wider range of Gram-positive and Gram-negative bacteria, totaling 23 genera, with more than one species or strain. While most of the bacteria tested were not affected by these β-lactams, a few important pathogenic genera, such as *Staphylococcus*, *Micrococcus* and *Bacillus*, were affected [69,73,74,75,76].

**Figure 4:**
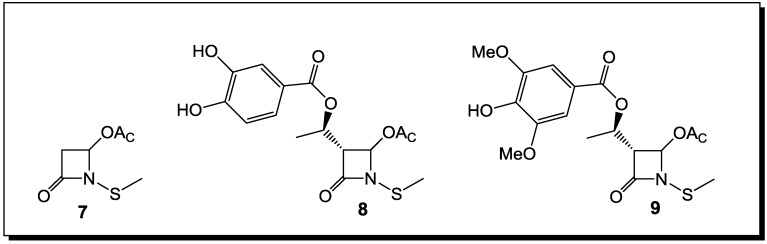
*N*-alkylthio β-lactams with activity against *S. aureus* infection associated with cystic fibrosis.

Thus, the selection of bacteria affected by the β-lactams is extremely narrow. The latter are highly selective towards *Staphylococcus* spp. and *Bacillus* spp. Moreover, unlike penicillins, which inhibit cell wall crosslinking enzymes, *N*-thiolated lactams are characterized by a bacteriostatic activity and act through a different mechanism of action [76]. While how these *N*-thiolated β-lactams exert their microbiological effects is still not completely understood, they clearly affect cellular processes associated with coenzyme A and lipid biosynthesis [76]. Recently, the representatives of these monocyclic *N*-methylthio-azetidinones were even reported as selective inhibitors of histone deacetylases (HDACs) [77]. In that case, the presence of an *N*-methylthio group had a key role in providing to the new β-lactams a stringent isoform selectivity. Many staphylococcal infections are associated with the development of resistance to β-lactam antibiotics. This is particularly important in patients with chronic diseases, such as cystic fibrosis (CF), where persistent colonization by pathogenic bacteria occurs and the repeated use of antibacterial agents selects for specific resistant strains. A rise in *S. aureus* infections has been reported in CF patients, with an increase in the prevalence of highly virulent MRSA [78]. The addition of a polyphenolic moiety to the *N*-alkylthio β-lactams proved to be of importance for activity in case of CF [78] (Figure 4).

The presence of oxidative stress in CF due to an increased production of reactive oxygen species (ROS) and to an impaired antioxidant status, particularly during chronic pulmonary infections, points to new therapeutic possibilities in targeting anti-oxidant pathways [78]. Thus, the necessity for antioxidant properties in the structure of an antibacterial agents against CF led to the preparation of dual activity *N*-alkylthio β-lactams, in which the presence of phenolic residues on the hydroxyethyl side chain switched upon the antioxidant potency. Several of these phenolic *N*-alkylthio lactams have been tested against clinically isolated CF, with encouraging results for their antibacterial activity [78]. Improving further on the structures of *N*-methylthio-4-acetoxy-azetidinones with polyphenolic residues on the side chain and subsequent testing on MRSA strains isolated from CF patients will further establish the structure-activity relationships (SAR) of these dual-action compounds [78]. 

**Figure 5:**
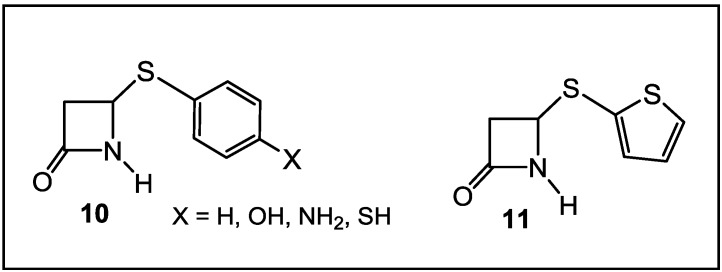
Arylthio-β-lactams active against *Moraxella catarrhalis* and *Mycobacterium tuberculosis*.

Monocyclic β-lactams, selective toward two phylogenetically unrelated microorganisms, *Moraxella catarrhalis* and *Mycobacterium tuberculosis* (Figure 5), have been also reported in the literature [79]. These compounds demonstrated intrinsic activity against serine β-lactamase producing and multiple (n = 6) β-lactamase producing *M. catarrhalis* clinical isolates. Both Kirby-Bauer disc diffusion assays and the Mininimal Inhibitory Concentration/Minimal bactericidal Concentration (MIC/MBC) ratio were used to screen compounds for antibacterial activity against non-β-lactamase producing American Type Culture Collection (ATCC) strains of *Escherichia coli*, *Pseudomonas aeruginosa*, *S. aureus* and *Enterococcus faecalis*, which are quality control strains routinely used for antimicrobial testing. They also represent a range of organisms, both Gram-positive and Gram-negative, that cause clinically important infections. None of the synthesized compounds affected the growth of *E. coli*, *P. aeruginosa*, *S. aureus* or *E. faecalis*. Further development on the SAR of these lactams will be reported in due time.

## 4. β-Lactams with Carboxylic Acid Bioisosteres as Leader Peptidase and Pilus Assembly Inhibitors

**Figure 6:**
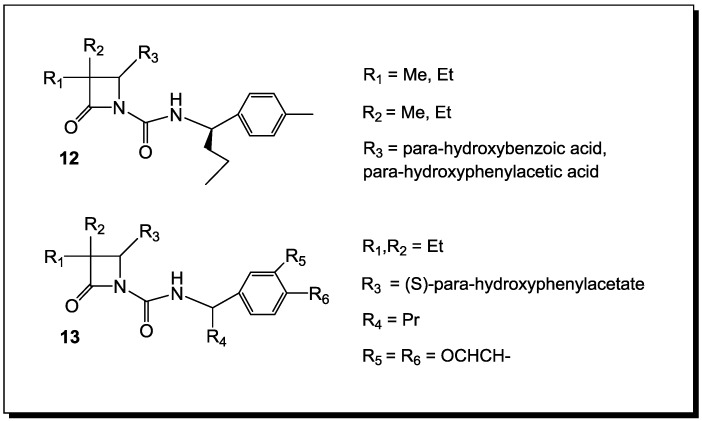
β-Lactams active against *Escherichia coli* leader peptidase.

β-Lactams have been shown to be inhibitors of *E. coli* leader peptidase [80,81]. This enzyme is an integral membrane protein, suggested to be a novel serine enzyme, which catalyzes the removal of the leader sequence as one of the last steps of the translocation of proteins across the cytoplasmic membrane [80]. The transport process is similar in both prokaryotes and eukaryotes. Monocyclic β-lactams (**12**, Figure 6) have been demonstrated to be time-dependent inhibitors of this enzyme [81]. The core structure of the evaluated compounds contains substituents at C3-position-short alkyl chains, a *para*-hydroxybenzoic acid at the C4-position and an N-1 substituent, thus making them similar to the inhibitory requirements for porcine pancreatic elastase (PPE) and human leukocyte elastase (HLE) (**13**). However, the C3-diethyl substituted β-lactams are not inhibitors of *E. coli* leader peptidase. 

**Figure 7:**
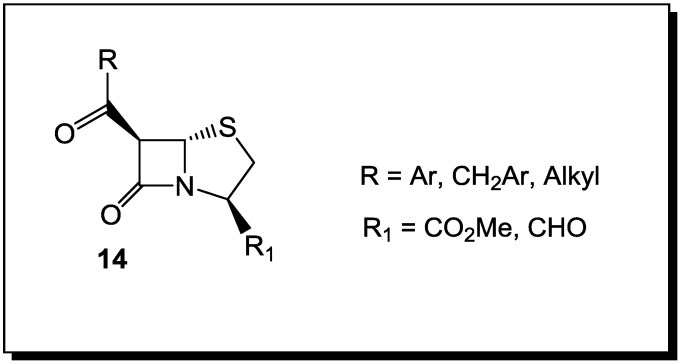
β-Lactams as inhibitors of bacterial pilus assembly.

In order to cause disease, bacteria need to adhere to host tissue. Many pathogenic species of bacteria develop pili—extracellular protein organelles in order to attach themselves to host epithelial cells. Pilus assembly is accomplished by periplasmic chaperons, which bring subunits to the outer cell membrane, where they are incorporated into the growing pilus [82]. Inhibition of pilus formation by a pilicide—a drug that can block this process—might be yet another addition to the existing arsenal of antimicrobial agents. Toward this end, penams with stereochemistry different than that of the original penicillins (**14**, Figure 7) have been designed to act as chaperone inhibitors [83].

This stereochemistry has been chosen in order to give these β-lactams the chance to withstand enzymatic degradation by penicillin-resistant bacteria. *In vitro*, the rigid β-lactam framework appears to mimic the peptides that are found to inhibit complex formation between PapD chaperone and the adhesin PapG [84]. However, *in situ* action awaits demonstration.

## 5. β-Lactams as Chemical Probes for Bacterial Enzymes

**Figure 8:**
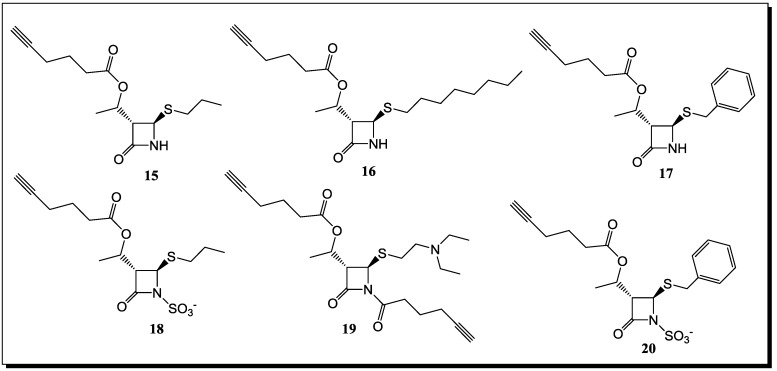
β-Lactam probes for identifying different bacterial drug targets.

Utilizing a chemical proteomic strategy called activity-based protein profiling (ABPP) for the identification of β-lactam binding enzymes under *in vivo* conditions has not only led to the labeling of the PBPs, which are known β-lactam targets, but also PBP unrelated enzymes. For the detection of the latter, several monocyclic β-lactams varying in substitution and reactivity were tested under *in vivo* conditions in three bacterial systems [85,86]. The monocyclic lactams (Figure 8) lacking an ionizable group at the lactam nitrogen do not label any PBPs, but bind to other important enzymes, such as β-ketoacyl acyl carrier protein III (KAS III) [87], a β-lactamase, a lipase acylhydrolase (Lip/Ac), a thiol-specific antioxidant (AhpC) [88] and the virulence-associated protein ClpP [89,90,91], confirming that it is possible to tune the selectivity of monocyclic β-lactams toward bacterial molecular targets by chemical modifications. The nucleophile, required for catalysis, in the active sites of these enzymes is either serine (β-lactamase, Lip/Ac and ClpP) or cysteine (KASIII and AhpC), which are likely to attack the β-lactam ring. ClpP is a central regulator of virulence, which is highly conserved in many pathogens, such as *S. aureus*. In fact, the labeling of recombinant *S. aureus* ClpP was achieved with the probe, **19** (Figure 8). Although this inhibition proved to be moderate, the scaffold, **19**, represents a good starting point for further optimization of potency [85].

All of these new lactam probe compounds were tested for growth inhibition of the corresponding bacteria, but had no effect on viability. However, the lack of antibiotic activity can be explained by their preference to bind predominantly targets that are not essential for viability, with the exception of KASIII. It could be that this enzyme is only partially inhibited by the lactam, which is not sufficient to observe a biological effect.

In addition to the labeling of the *B. licheniformis* β-lactamase with high efficiency, some of the β-lactam probes were also able to inhibit this enzyme. Probe **19** (Figure 8) turned out to be the most selective probe, with no off targets observed, even at a concentration of 50 μM in the background of the total proteome and a detection limit of 3 μM [85]. In addition, the more reactive Probe **19** exhibited an even lower detection limit of <0.5 μM, but showed labeling of additional targets at concentrations >3 μM. Significant inhibition by Probes **15**, **17** and **19** could be obtained with corresponding IC_50_-values of 2.5, two and <0.5 μM, respectively. The long-chain hydrophobic Compound **16**, regardless of not being soluble at concentrations above 25 μM in the lactamase assay buffer, revealed still significant inhibition below 10 μM. In addition, a different recombinant β-lactamase from *B. subtilis* (PBP4*) also revealed specific labeling with all probes, except for the sulfonylated Compound **18** (Figure 8). The latter is especially intriguing, since all of the clinically relevant β-lactamase inhibitors contain an ionizable group at the lactam nitrogen. In general, these results emphasize the utility of β-lactams for the identification of β-lactam binding enzymes and study their function and regulation *in vivo.* Future studies with β-lactam resistant pathogens will show whether these probes can help to restore or increase antibiotic susceptibility and help to overcome the pressing problem of antibiotic resistance.

## 6. Conclusions

Currently, all of the clinically relevant β-lactam antibiotics (penicillin-like bicyclic structures and monobactams) and the β-lactamase inhibitors have a negatively charged substituent (*i.e*., the C3 carboxylate of penicillin or a suitable mimic) necessary for recognition in the active site of PBPs and β-lactamases. The antimicrobial activity of monocyclic β-lactams lacking the ionizable group at N1 have demonstrated that new antibiotic leads could be identified for the treatment of specific drug-resistant, β-lactamase producing bacterial pathogens without the need for β-lactamase inhibition. More studies on the chemical nature of the interaction of these β-lactams with different from “usual” molecular targets could lead to the development of β-lactam-based inhibitors with pharmacological efficacy toward very important pathogens. Now, in the early twenty-first century, the search for new antibacterial agents and novel strategies for combating bacterial resistance remains urgent and extremely challenging. Our developing understanding of the “nontraditional” roles of the β-lactams as inhibitors of different bacterial enzymes is leading us to their use as platforms for the design of new therapeutic agents.
